# HERV-W upregulation expression in bipolar disorder and schizophrenia: unraveling potential links to systemic immune/inflammation status

**DOI:** 10.1186/s12977-024-00640-3

**Published:** 2024-04-22

**Authors:** Sara Coelho Rangel, Michelly Damasceno da Silva, Décio Gilberto Natrielli Filho, Samuel Nascimento Santos, Jonatas Bussador do Amaral, Jefferson Russo Victor, Kevin Cezar Nascimento Silva, Izabela Dorota Tuleta, Carolina Nunes França, Marina Tiemi Shio, Lucas Melo Neves, André Luis Lacerda Bachi, Luiz Henrique da Silva Nali

**Affiliations:** 1 Post-graduation Program in Health Sciences, Santo Amaro University, Rua Isabel Schmitt, 540, São Paulo, Brazil; 2 Hospital Escola Wladimir Arruda– Departamento de Psiquiatria– Santo Amaro University, Rua Prof. Enéas de Siqueira Neto, 340, São Paulo, Brazil; 3 Ent Research Lab, Department of Otorhinolaryngology-Head and Neck Surgery, Federal University of Sao Paulo, São Paulo, Brazil; 4 Department of Medicine-Cardiology, Albert Einstein College of Medicine, New York, EUA USA

**Keywords:** Bipolar Disorder, Schizophrenia, Human Endogenous Retrovirus, Expression, Cytokines, Inflammation

## Abstract

**Background:**

Bipolar disorder (BD) and schizophrenia (SZ) are the two main mental disorders with unknown etiology that significantly impact individuals’ quality of life. The potential pro-inflammatory role in their pathogenesis is postulated and Human Endogenous Retrovirus W (HERV-W) is an emerging candidate to modulate this pathogenic finding. HERVs, ancient retroviruses in the human genome, may play roles in inflammation and disease pathogenesis. Despite HERVs’ involvement in autoimmune diseases, their influence on mental disorders remains underexplored. Therefore, the aim of this study was to assess the level of HERV-W-env expression and the systemic inflammatory profile through the concentration of IL-2, IL-4, IL-6, IL-10, TNF-α and INF-γ cytokines in BD and SZ patients.

**Results:**

All participants showed HERV-W-env expression, but its expression was higher in mental disorder patients (*p* < 0.01) than in control. When separated, SZ individuals exhibited higher HERV-W expression than the control group (*p* < 0.01). Higher serum levels of TNF-α and IL-10 were found in BD (*p* = 0.0001 and *p* = 0.001, respectively) and SZ (*p* = 0.01) and *p* = 0.01, respectively) than in the control group, while SZ showed decreased levels IFN-γ and IL-2 as compared to controls (*p* = 0.05) and BD patients (*p* = 0.05), respectively. Higher TNF-α/IL-4 and TNF-α/IL-10 ratios, and lower IFN-γ/IL-10 were observed in BD and SZ patients than controls. Significant negative correlation between HERV-W-env expression and IL-10 (*r*=-0.47 *p* < 0.05), as well as positive correlations between HERV-W-env expression and TNF-α/IL-10 or IFN-γ/IL-10 ratios (*r* = 0.48 *p* < 0.05 and *r* = 0.46 *p* < 0.05, respectively) were found in BD patients.

**Conclusion:**

These findings suggest not only a potential link between HERV-W-env expression both in BD and SZ, but also a possible involvement of systemic inflammatory status in BD patients.

## Background

Bipolar disorder (BD) and Schizophrenia (SZ) are two main frequent and severe mental disorders that significantly compromise the quality of life and the social conditions of affected individuals [1]. Both disorders do not present any specific etiology, but genetic and environmental factors may be associated with the disease onset and clinical evolution of the disease [2, 3]. Despite the unknown etiology of the disease, it is utmost to emphasize the possible involvement of systemic pro-inflammatory status in the context of the disease’s pathogenesis [4, 5].

However, triggering this inflammatory response profile and sustaining this condition in both these diseases is still poorly understood. In this sense, Human Endogenous Retroviruses (HERV) may be highlighted not only as a potential candidate for promoting inflammatory response [6, 7], but might also be associated with these disease’s pathogenesis [8, 9].

HERVs are ancient retroviruses that have become integral components of the human genome, which originated from infections in our ancestor’s germline cells millions of years ago [10–12]. These retroviruses have been transmitted and perpetuated initially by horizontal transmission [13] and later inherited by Mendelian way [14], and today we know that HERVs compose 8% of the human genome [10–12, 15]. Over generations, retrotransposition events have contributed to the genomic diversity of these elements within the genome. However, HERVs have undergone mutations, resulting in their silencing by the presence of stop codon within the coding region, isolated genes/Long Terminal Repeats (LTRs) and incomplete sequences, which resulted in their inability to replicate [16]. Nevertheless, HERVs can still exhibit expression, and the virions may be formed through the combination of retroviral genes from various loci within the genome [13, 17–19].

Notably, these retroviruses play crucial roles in human physiology, e.g. the HERV-W-env protein, also known as Syncytin-1, assumes pivotal participation in human placentation by mediating the fusion of Syncytiotrophoblast during early nidation and placenta formation [20]. Additionally, the LTRs of HERVs may serve as promoters for human genes [21, 22]. Unfortunately, HERVs are also linked to the development of autoimmune diseases, such as Multiple Sclerosis, as it has been described in the past 30 years [23–30]. Interestingly, HERVs may promote an inflammatory response, and some pieces of evidence also point out that the inflammatory response may trigger HERV expression [6, 7], suggesting a looping between them.

HERVs have been postulated to play a role in BD and SZ diseases since there was a higher level of HERVs expression in brains Cerebrospinal fluid (CSF) and Peripheral Blood Mononuclear cells (PBMC) from patients with these diseases [31, 32]. Furthermore, higher concentration of some pro-inflammatory cytokines associated with HERV-W antigenemia [33] and direct role of HERV in the pathogenic reduction of neuronal density in the hippocampus region and expressive changes of dendritic morphologic were found in SZ patients [34]. Therefore, this evidence indicates possible and dynamic mechanisms for the role of HERVs, especially HERV-W, in BD and SZ’s pathogenesis.

Regarding systemic immune/inflammatory status in mental diseases, it was documented that the balance between some pro-inflammatory cytokines, such as TNF-α and IFN-γ, and anti-inflammatory cytokines, such as IL-4 and IL-10, which are involved in the T-helper type 1 (Th1) and T-helper type 2 (Th2) profiles, plays a corollary role in this mental disorders as BD [35] and SZ [36].

Although compelling evidence points out that HERVs might be associated with the pathogenesis of mental disorders, such as BD and SZ, the interplay between HERV-W-env expression in those patients and its systemic inflammatory status is still poorly understood. Based on this, here, we aimed to investigate both the HERV-W-env expression and the systemic inflammatory status in BD and SZ patients.

## Methods

### Study population

Patients with mental disorders (MD) (*n* = 48) were separated into two groups according to their diagnosis in Schizophrenia (SZ, *n* = 24) and Bipolar Disorder (BD, *n* = 24) groups. Another group with healthy individuals, paired by age without any previous report of autoimmune disease and mental disorders in the family, was included as a control group (*n* = 46). Sociodemographic data were collected from all volunteers through questionnaires. MD patients had previous diagnosis and were followed in the psychiatric outpatient clinic of Universidade Santo Amaro and in the Centro de Apoio de Atenção Psicossocial (CAPS), both located in the south region of São Paulo city, Brazil. Mini International Neuropsychiatric Interview (MINI) [37, 38] was used to confirm the diagnosis. Only patients out of the mania phase were included in the BD group. Symptoms of depression were assessed by Montgomery–Åsberg Depression Rating Scale [39] and symptoms of mania by Young Mania Rating Scale [40]. For the SZ group we recruited only patients who were not in a psychosis event. The study was approved by the ethical committee of Universidade Santo Amaro under protocol # 5.469.700. It is worth mentioning that the study was performed in agreement with the Declaration of Helsinki.

### Blood sample collection and preparation

Blood samples were collected in tubes containing the anticoagulant EDTA for peripheral blood mononuclear cells (PBMCs) obtention, which was used to perform the molecular analysis, and in gel-barrier dry tubes for serum obtention, which was used to determine the systemic cytokine concentration.

PBMCs were obtained by Ficoll-HyPaque protocol and RNA was extracted by Trizol-chloroform Method. Briefly, Ficoll-Hypaque was added into whole blood in 1:1 proportion and centrifuged at 800 g for 20 min. Afterward, the upper solution (PBMC) was collected and washed repeatedly with sterile PBS and centrifugation until the removal of residual erythrocytes was removed entirely. Finally, cell pellet containing exclusively the PBMCs undergoing RNA extraction as follows: 1mL of Trizol was added into each of cell pellet samples and up-down was performed until complete homogenization, then 200µL of chloroform was added and samples were centrifuged at 10.000 g at -4^o^C. The upper phase was completely removed (around 600 µL) and then RNA was precipitated with Isopropanol 100% and washed twice with 70% Ethanol. RNA was resuspended in 40 µL H_2_O-Nuclease free. Rigorous decontamination of genomic DNA was performed with DNA-free turbo (Ambion). The absence of contaminant genomic DNA was confirmed by Real-Time PCR with primers complementary to the GAPDH gene with the absence of Reverse Transcriptase. RNA was quantified and then stored at -80^o^C. Around 150ng of RNA was used to synthesize the cDNA with a High-Capacity Reverse Transcription Kit.

### HERV-W detection and relative quantification analysis

We used primers complementary to HERV-W-env [41] and GAPDH as housekeeping genes [42]. The RT-PCR mix included 0.1 µM of each primer and 1x of PCR Master Mix Sybr-Green one step (Merck). The cycling conditions for HERV-W were: 50oC for 2 min, 95oC for 10 min followed by 40 cycles of 95oC for 1 min, 50oC for 45 s and 60oC for 1 min. For GAPDH assay the cycling conditions were: 50oC for 2 min, 95oC for 10 min followed by 40 cycles of 95oC for 1 min and 60oC for 1 min. In both assays a previous step was added of 37oC for 30 min for cDNA synthesis and a final cycle to determine the melting temperature of the samples (55oC to 95oC). HERV activity expression was evaluated qualitatively (absence/presence) and quantitatively (level of expression). The level of expression was determined by the 2-ΔΔCt method where ΔCt = (HERV Ct- GAPDH Endogenous Control Ct)– (Average of ΔCt of all controls), and the results were represented as fold changes. In all cases, samples were considered positive for HERVs if the melting curve was the same or ± 0.3oC distinct from the control samples and therefore included in the relative quantification analysis.

### Determination of systemic cytokine concentration

Serum concentrations of the cytokines IL-2, IL-4, IL-6, IL-10, TNF-α and INF-γ were determined by using the ELISA commercial kits (ThermoFisher), following the manufacturer’s instructions. The concentration of each cytokine was calculated through an appropriate standard curve that presented a correlation coefficient from 0.95 to 0.99, with coefficients of variance intra-assay varying from 2,5 to 4% and from 8 to 10% in inter-assay.

### Statistical analysis

The sample size was collected by convenience. Descriptive data was obtained, and for the comparison of scores between different groups, both parametric and non-parametric tests were employed according to the normality distribution of the data. The tests used were as follows: The normality test was performed using the Shapiro-Wilk test, and the homogeneity of variance was evaluated using the Levene test. Mann-Whitney test was used to analyze HERV-W-env expression between MD and control groups, whereas the Kruskal-Wallis test was used to assess the differences between the BD, SZ, and control groups. In addition, Spearman’s rank coefficient correlation test was also applied. All tests were conducted under the assumption of a first-type error probability (alpha) of 5% (*p* < 0.05).

## Results

### Clinical and demographic findings of the volunteers

Table 1 summarizes the demographic characteristics of the volunteers included in the present study.

**Table 1:** Demographic characteristics of the volunteers enrolled in the study

	BD (*n* = 24)	SZ (*n* = 24)	HG (*n* = 46)
Sex F/M	21/03	07/17	43/03
Age (mean and SD)	41 ± 13,1	42 ± 13,5	41,77 ± 7,2
MADRS (mean and SD)	14 ± 4	Not applicable	Not applicable
YOUNG (mean and SD)	6 ± 2	Not applicable	Not applicable
Employment status (n/%)	Yes (10/41.6) No (14/58.3)	Yes (01/4.1) No (23/95.9)	Yes (43/93.5) No (03/6.5)
Medications related to MD (n/%)	Alprazolam (1/4.1) Biperiden (1/4.1) Carbamazepine (2/8.3) Lithium carbonate (11/45.8) Chlorpromazine (2/8.3) Diazepam (1/4.1) Fluoxetine (1/4.1) Haloperidol (1/4.1) Lurasidone (1/4.1) Pregabalin (1/4.1) Quetiapine (3/12.5) Rispiridone (8/33.3) Sodium volproate (9/37.5)	Biperiden (4/16.6) Lithium carbonate (2/8.3) Clonazepam (3/12.5) Fluoxetine (4/16.6) Chlorpromazine (3/12.5) Clozapine (1/4.1) Haloperidol (3/12.5) Olanzapine (1/4.1) Rispiridone (7/29.1) Sodium volprate (4/16.6)	Not applicable
Family history of MD (n/%)	Yes (14/58.3) No (10/41.7)	Yes (11/45.3) No (13/54.7)	Yes (0/0) No (46/100)

Legend: F/M: Female/Male; SD: Standard deviation; BD: Bipolar Disorder; SZ: Schizophrenia; HG: Healthy individuals’ group; MD: Mental disorders; MADRS: Score on Montgomery–Åsberg Depression Rating Scale; YOUNG: Score on the Young Mania Rating Scale.

In addition to the demographic findings, it is noteworthy to mention that individuals in the BD group received a confirmed diagnosis at an average age of 26 ± 11.3 years, and the SZ group received a confirmed diagnosis at a similar average age (27 ± 10.9 years). In addition, both groups have been diagnosed for more than 15 years. It is essential to mention that most of the patients enrolled did not exhibit any other comorbidities, with only 8 (33%) in the BD group and 7 (25%) in the SZ group presenting additional inflammatory disorders, such as hypertension and dyslipidemia.

### HERV-W-envexpression is upregulated in SZ patients

**Fig. 1:**
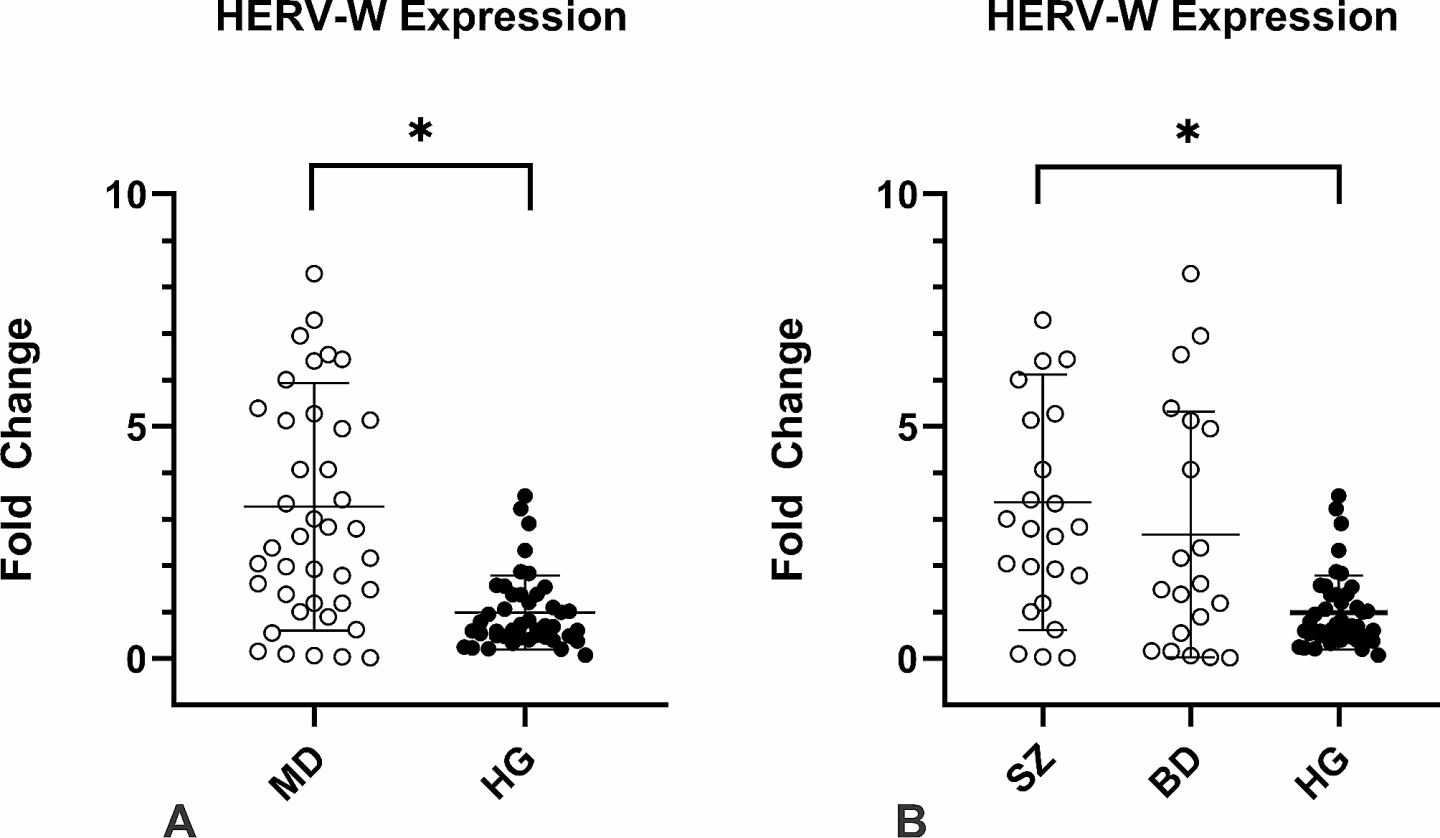
HERV-W env relative expression in the individuals enrolled in the study. Figure 1 A: MD patients presented higher HERV-W expression *p < 0.01; Fig. 1B: HERV-W expression levels were assessed in MD individuals according to each group. SZ patients showed significantly higher levels of HERV-W expression than CG *p < 0.01, Mann-Whitney`s test

All participants of the study showed expression of HERV-W-env. However, the expression in MD patients was 3.3-fold higher on average than the control group (*p* < 0.01, Fig. 1A). When analyzing the HERV-W-env expression levels in the SZ and BD groups separately, the SZ group presented 3.3-fold higher on average than the control group (*p* < 0.01, Fig. 1B), whilst the BD group did not show significant differences in the HERV-W-env expression as compared to the control group (*p* = 0.54, Fig. 1B). In addition we have performed the analysis of HERV-W expression according to the therapeutic scheme, however no significant difference was found in any analysis.

### BD and SZ patients present higher concentration of some systemic cytokines

**Fig. 2:**
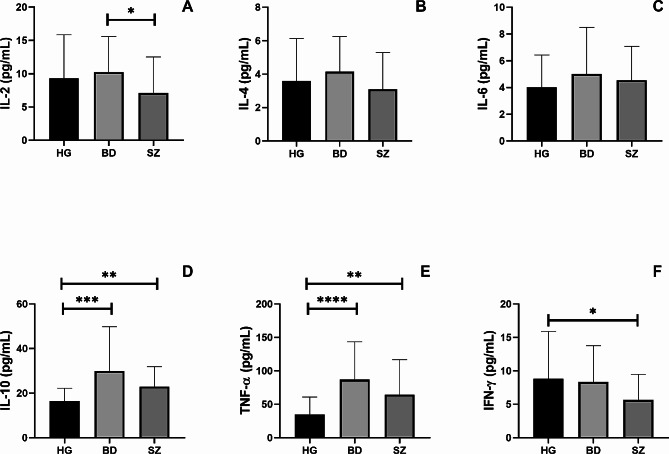
Overall pro and anti-inflammatory cytokines concentration analysis in the serum of individuals enrolled in the study. Legend: BD = Bipolar Disorder Group, SZ = Schizophrenia Group, HG = Healthy individuals Group *= *p* < 0.05, **=*p* < 0.01, ***=*p* < 0.001, ****p < 0.0001

Figure 2 shows the systemic cytokine levels of IL-2 (Fig. 2A), IL-4 (Fig. 2B), IL-6 (Fig. 2C), IL-10 (Fig. 2D), TNF-α (Fig. 2E), and IFN-γ (Fig. 2F) in the volunteer groups. Both BD and SZ groups presented higher serum concentrations of IL-10 (*p* = 0.001 and *p* = 0.01, respectively) and TNF-α (*p* = 0.0001 and *p* = 0.01, respectively) than those observed in the control group. In addition, the SZ group showed lower circulating levels of IL-2 and IFN-γ than the BD group (*p* = 0.05) and the control group (*p* = 0.05), respectively.

**Fig. 3:**
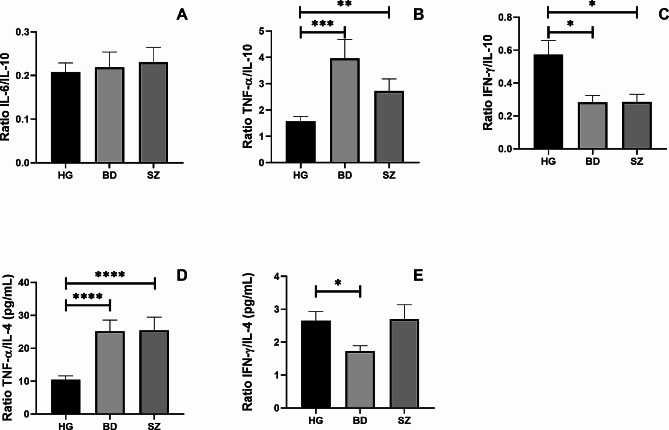
The ratio between pro-inflammatory and anti-inflammatory cytokines in HG, BD and SZ groups. The ratio between the concentrations of the pro-inflammatory cytokines IL-6 **(A)**, TNF-α **(B)**, and IFN-γ **(C)** and the anti-inflammatory cytokine IL-10 and the ratio between the concentrations of the pro-inflammatory cytokines TNF-α **(D)**, IFN-γ **(E)** and the anti-inflammatory cytokine IL-4 in the plasma of patients enrolled in the study. Legend: BD = Bipolar Disorder Group, SZ = Schizophrenia Group, HG = Healthy individuals Group *= *p* < 0.05, **=*p* < 0.01, ***=*p* < 0.001, ****p < 0.0001

**Fig. 4:**
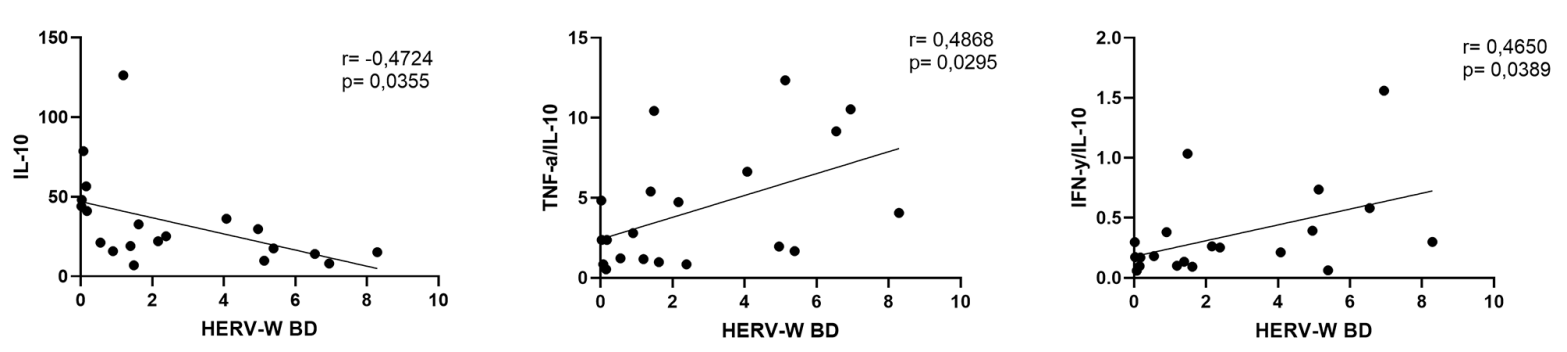
Correlation between HERV-W expression and cytokines to BD group. Correlation between IL-10 and HERV-W **(A)**, Correlation between the ratio of TNF-α/IL-10 with HERV-W **(B)**, Correlation between the ratio of IFN-*γ*/IL-10 with HERV-W **(C)**

Additionally, Fig. 3 shows the ratio between the circulating levels of IL-6/IL-10 (Fig. 3A), TNF-α/IL-10 (Fig. 3B), IFN-γ/IL-10 (Fig. 3C), TNF-α/IL-4 (Fig. 3D), and IFN-γ/IL-4 (Fig. 4E) was also assessed. Higher TNF-α/IL-4 (*p* = 0.0001) and TNF-α/IL-10 (*p* = 0.001) ratios, as well as lower IFN-γ/IL-10 ratio (*p* = 0.05), were found in BD and SZ groups than in the control group. In addition, a lower IFN-γ/IL-4 ratio was observed in the BD group than in the control group (*p* = 0.05).

### HERV-W-env expression correlates with the pro-inflammatory profile in BD patients

Figure 4 shows the significant results obtained in the Spearman coefficient correlation analysis. Exclusively in the BD group, it was observed a significant negative correlation (*r*=-0.4724) between the levels of HERV-W-env expression and serum IL-10 (Fig. 4A), as well as significant positive correlations between the HERV-W-env expression levels and TNF-α/IL-10 (*r* = 0.4868) (Fig. 4B) or IFN-γ/IL-10 (*r* = 0.4650) (Fig. 4C) ratios.

## Discussion

Here, we have described a higher HERV-W-env expression in PBMCs obtained from MD patients compared to healthy individuals, and, specifically, the SZ group showed that this increase was 3.3-fold higher than HG (*p* < 0.01). This finding is not only in touch with previous studies that described higher levels of HERV-W expression in SZ patients [43], but can also provide additional evidence supporting the involvement of altered HERV-W-env activity in SZ patients. Moreover, and in an exciting way, SZ patients presented higher serum TNF-α concentrations than the control group, despite this finding not being positively correlated with HERV-W expression nor the pro- and anti-inflammatory cytokines ratios. This result not only corroborates the suggestion that the systemic inflammatory status has an essential role in SZ, but also might indicate that this condition was not closely associated with the HERV-W expression, even though its expression was also increased in those patients. Based on these facts, we can putatively suggest that systemic inflammatory status and HERV-W expression components may show distinct pathogenic roles in this disease. Previous reports in the literature have pointed out a direct involvement of neuronal pathological modifications with HERV-W-env expression and, particularly, some of these findings should be highlighted: neuronal apoptosis induction [44], structural and functional abnormalities in dopaminergic neurons that stimulates substantially the production of dopamine through Dopamine Receptor D2 (DRD2) and leads to alteration in sodium and calcium influx, which suggests a pivotal role of HERV-W not only in neuronal pathophysiology [45] but also in the reduction of hippocampal neurons density and the alteration of its dendritic and perikaryon morphology [46]. Although these previous data are very interesting in the context of HER-W expression and the nervous system, it is noteworthy to cite that our results were associated with the HERV-W-env expression in PBMC and not specifically concerning the central nervous system (CNS).

In a different way to SZ patients, the BD patients did not show significant differences in the HERV-W-env expression in PBMC as compared to the control group. This finding is in contrast with previous reports in the literature since it has been demonstrated higher levels of HERV-W expression both systemically [43] and in the brain [47] of BD patients. Even though we cannot affirm, some hypotheses can help us to understand this lack of a significant difference in HERV-W-env expression between BD and control groups found here: (i) since the HERV-W-env expression levels could putatively be impacted by different clinical manifestations in BD, it would be expected that BD patients in euthymic conditions could present a reduction on the HERV-W-env expression levels, however, it is paramount to mention that, until now, the dynamics of HERV expression in BD patients was not fully understood, thus a remarkable variation in their expression can also be presented in euthymic condition; and: (ii) despite no significant difference was found, a tendency for it could be observed (*p* = 0.054) and maybe this lack of statistically significant difference could be related to the number of BD patients enrolled in the study.

Previous findings report that both SZ and BD patients are associated with the deregulation of the systemic cytokine’s concentration. The main hypothesis is that the inflammatory condition may interfere specially in the blood and brain barrier permeability [48, 49]. It is supposed that in both BD and SZ, a pro-inflammatory profile is present and, chronically, could promote pathological modification in the CNS way before the disease’s onset. In this sense, it is known that not only genetic background added to the fundamental environmental factors is necessary to deregulate the balance of the immune-inflammatory responses [50] but also, as previously cited, that HERVs may elicit the inflammation in both physiological and pathological conditions [7, 51, 52]. Taken together, these pieces of information can corroborate our findings in which BD patients presented not only higher circulating TNF-α levels, a well-known pro-inflammatory cytokine associated with Th1 immune profile, than the control group but also a significant negative correlation between the levels of HERV-W-env expression and IL-10, a classical anti-inflammatory cytokine, besides significant positive correlations with TNF-α/IL-10 and IFN-γ/IL-10 ratios. Based on these data, it is clear that BD patients presented a prominent systemic pro-inflammatory status, which agrees with previous reports [53–55]. At this point, it is paramount to highlight that the ratio assessment has been considered as an accurate measure concerning the balance of pro- and anti-inflammatory cytokines in different contexts, including in MD patients [56]. Moreover, it is worth also mentioning that, although the pro-inflammatory status is present in BD patients, the triggers involved in this condition are yet to be found [57]. Thus, our findings can putatively suggest that HERV-W-env could be a potential player in this context. However, this hypothesis requires further investigation.

Beyond these findings, it is also of utmost importance to highlight that, regardless of the association with HERV-W-env expression, both BP and SZ groups showed a higher TNF-α/IL-4 ratio, which together with the higher TNF-α/IL-10 ratio, than the control group, which allows us to suggest that, in general, the T-help immune response was towards to Th1 profile, since there is a consensus that both TNF-α and IFN-γ are related to the Th1 immune profile, whereas the IL-4 is a classical cytokine of Th2 immune profile and IL-10 is closely associated with T regulatory (T-reg) immune profile [58]. In fact, according to the literature, increased TNF-α/IL-4 and IFN-γ/IL-4 ratios were verified in BP patients during manic episodes as compared with normal controls [59], and a relative predominance of the Th2 immune profile was evidenced in patients with acute exacerbation of schizophrenia [36]. Despite an imbalance in the Th1/Th2/Treg immune profile could be related to some worse outcomes both in BP and SZ patients, the results observed here allow us to putatively suggest that a “regulated” Th1 immune profile was present in the BP and SZ individuals enrolled in the present study since they showed not only higher circulating IL-10 levels but also lower IFN-γ/IL-10 ratio than the control group. In addition, the BP group also showed a significant reduction in the IFN-γ/IL-4 ratio compared to the control group, which corroborates our suggestion that although the Th1 immune response was predominant, this profile was not exacerbated at this point.

Among other limitations of the study formerly cited, our study presents another important limitation that should be mentioned since none of the patients enrolled in the study were under clinical episodes of BD and SZ in the sampling time, which might underestimate the level of HERV-W-env expression and cytokine concentration in thisstudy. Also, we were not able to gather enough patients to properly compare the levels of HERV-W-env expression and the systemic cytokine concentration according to the therapeutic scheme.

In summary, we have described high levels of HERV-W-env expression in SZ patients, which generally did not show a close association with the systemic cytokine levels. Therefore, in those patients, whereas the HERV-W-env may be related to SZ pathogenesis, their interplay with systemic inflammatory status was not evidenced, which allows us to suppose that HERV could act directly in a pathological manner, causing cellular changes capable of interfering with neuronal functioning, and not necessarily immune-inflammatory mediated. On the other hand, BD seems to present a distinct profile since a close association with the systemic inflammatory status was evidenced in those patients. Even though the HERV-W-env expression was not significantly higher in BD patients (*p* = 0.054), significant positive correlations between HERV-W-env expression and TNF-α/IL-10 and IFN-γ/IL-10 ratios, as well as significant negative correlation observed between the level of HERV-W-env expression and circulating IL-10, were found. Based on these findings, it is reasonable to consider that HERV-W-env may be modulating the inflammatory conditions of BD patients, which might propose a distinct pathogenic mechanism for this disease in contrast to SZ patients.

Finally, although significant findings have continually been described, subsequent studies should focus on understanding the natural role of HERVs in the systemic inflammatory profile in these diseases in order not only to identify possible interplay pathways between them in this context as well as to investigate whether HERV-W-env could represent a possible target candidate for treatment of both diseases. Additionally, and equally important, a comprehensive analysis of distinct HERV families upregulated in MD patients should also be a priority to improve the understanding of the dynamics of these retroelements’ expression in the MD pathogenesis.

## Data Availability

No datasets were generated or analysed during the current study.
